# Polypyrrole with Phosphor Tungsten Acid and Carbide-Derived Carbon: Change of Solvent in Electropolymerization and Linear Actuation

**DOI:** 10.3390/ma14216302

**Published:** 2021-10-22

**Authors:** Chau B. Tran, Zane Zondaka, Quoc Bao Le, Bharath Kumar Velmurugan, Rudolf Kiefer

**Affiliations:** 1 Faculty of Applied Sciences, Ton Duc Thang University, Ho Chi Minh City 700000, Vietnam; tranboichau@tdtu.edu.vn; 2 Intelligent Materials and Systems Lab, Faculty of Science and Technology, University of Tartu, Nooruse 1, 50411 Tartu, Estonia; zane.zondaka@ut.ee; 3 Conducting Polymers in Composites and Applications Research Group, Faculty of Applied Sciences, Ton Duc Thang University, Ho Chi Minh City 700000, Vietnam; lequocbao@tdtu.edu.vn; 4 Department of Medical Laboratory Science and Biotechnology, Asia University, Taichung 413, Taiwan; bharathvel@gmail.com

**Keywords:** solvent change, electropolymerization, linear actuation, PPyPT, PPyCDC

## Abstract

Linear actuators based on polypyrrole (PPy) are envisaged to have only one ion that triggers the actuation direction, either at oxidation (anion-driven) or at reduction (cation-driven). PPy doped with dodecylbenzenesulfonate (PPy/DBS) is the most common applied conducting polymer having cation-driven actuation in aqueous solvent and mainly anion-driven actuation in an organic electrolyte. It is somehow desired to have an actuator that is independent of the applied solvent in the same actuation direction. In this research we made PPy/DBS with the addition of phosphorus tungsten acid, forming PPyPT films, as well with included carbide derived carbon (CDC) resulting in PPyCDC films. The solvent in electropolymerization was changed from an aqueous ethylene glycol mixture to pure EG forming PPyPT-EG and PPyCDC-EG composites. Our goal in this study was to investigate the linear actuation properties of PPy composites applying sodium perchlorate in aqueous (NaClO_4_-aq) and propylene carbonate (NaClO_4_-PC) electrolytes. Cyclic voltammetry and square potential steps in combination with electro-chemo-mechanical-deformation (ECMD) measurements of PPy composite films were performed. The PPyPT and PPyCDC had mixed ion-actuation in NaClO_4_-PC while in NaClO4-aq expansion at reduction (cation-driven) was observed. Those novel PPy composites electropolymerized in EG solvent showed independently which solvent applied mainly expansion at reduction (cation-driven actuator). Chronopotentiometric measurements were performed on all composites, revealing excellent specific capacitance up to 190 F g^−1^ for PPyCDC-EG (best capacitance retention of 90 % after 1000 cycles) and 130 F g^−1^ for PPyPT-EG in aqueous electrolyte. The films were characterized by scanning electron microscopy (SEM), Raman, Fourier-transform infrared (FTIR) and energy dispersive X-ray spectroscopy (EDX).

## 1. Introduction

PPy doped with DBS^−^ is one of the best studied types of conducting polymer materials, found in various formations in micro-fabrication [1] such as micro-robotic devices [2,3] biomedical applications [4], biochips to trigger cell growth over actuation [5] and, recently, smart textile fabrications [6]. Addition of charged molecules such as polyoxometalates (Keggin type [7], phosphotungstic acid (PTA, PW_12_O_40_^3−^)) forming PPyDBS-PT composites revealed strain in aqueous electrolyte in range of 5.2% [8]. Additional incorporation of meso-porous carbide-derived carbon (CDC) particles forming PPyDBS-PT-CDC (PPyCDC) linear films [9] had strain in the range of 12% while PPyDBS linear actuators strain values varied at 4–6% strain depending on the choice of electrolyte applied [10]. In general PPyDBS (as well with additional molecules such as PTA and CDC) show main expansion at reduction in aqueous electrolytes with so called cation-driven actuators (Equation (1)).

(1) $$[\left({\mathrm{PPy}}^{n+}\right)\left({\mathrm{MA}}^{-}{)}_{n}\right]+\;n\left(C^{+}\right)+m\left(S\right)+\;n\left(e^{-}\right)\overset{\mathrm{red}}{\underset{\mathrm{ox}}{⇌}}\;[\mathrm{PPy}{)}^{0}{\left(A^{-}\right)}_{n}{\left(C^{+}\right)}_{n}{\left(S\right)}_{m}$$

The left side of Equation (1) shows oxidized PPy with embedded immobile macro-anons MA^−^ which are compensated overcharged PPy^n+^. At reduction (right side of Equation (1)) PPy is reduced and the negative charge provided by immobile macro-anions (MA^−^) is compensated with solvated cations of the surrounding electrolyte.

In organic electrolytes it was found that the actuation direction changed [11] due to the influence of organic solvent on incorporated macro-anions (DBS^−^ PT^4−^) having a low dissociation degree [12], leading to ingress of anions (anion-driven) at oxidation [13], following Equation (2).

(2) $$[{\left(\mathrm{PPy}\right)}^{n+}{\left({\mathrm{MA}}^{-}\right)}_{m}{\left(C^{+}\right)}_{m}{\left(\mathrm{PC}\right)}_{\left(o+p\right)}{\left(A^{-}\right)}_{n}+n{\left(e^{-}\right)}_{\mathrm{metal}}\overset{\mathrm{red}}{\underset{\mathrm{ox}}{⇌}}[\left(\mathrm{PPy}\right)\;{\left({\mathrm{MA}}^{-}\right)}_{m}{\left(C^{+}\right)}_{m}\left(\mathrm{PC}{)}_{o}\right]+{\;\mathrm{nA}}^{-}+\;\mathrm{pPC}$$

The macro-anions combined with cations are functionalized as neutral units which do not influence the actuation properties of PPy/DBS based composites in organic electrolytes. Previous research [14] of PPy/DBS showed by applying different electrolytes in propylene carbonate main expansion at oxidation but also small parts of expansion at reduction was discovered. The expansion at reduction in organic electrolyte was found to be the case of PPy films having some incorporated anions at oxidation that stayed immobile inside the PPy films, leading to expansion at reduction. Such tendencies were shown in previous research [15] of typical anion-driven actuators of PPy/CF_3_SO_3_ films.

It is somehow desired to have only one actuation direction independent of what solvent is applied. Therefore, our goal was to investigate at first how changes in electropolymerization affecting the novel PPy composite films and as a second goal how the electrolyte and solvent influencing the linear actuation in direction as well as in achieved strain.

Most research was made using polyethylene glycol (PEG) rather than ethylene glycol in combination with PPy. Such an approach was an example of chemical polymerization forming PPy-PEG particles and did reveal good dispersibility and stability in aqueous and organic electrolytes [16]. There were few actuation studies made [17]; there was one example of PPy-PEG in bilayer studies where it was shown that the water adsorption properties increased with the amount of PEG applied in electropolymerization with increasing electronic conductivity [18]. At high PEG concentration a decrease of bending displacement was observed [19]. Other research of PPy inclusion of PEG revealed that the hydrophilic characteristics increased [20] as well as discovery of a certain affinity of PEG to metallic cations rather than anions [21].

Electrochemical techniques such as cyclic voltammetry and square potential steps at applied frequencies from 0.0025 to 0.1 Hz were made of PPy composites in combination with ECMD measurements to investigate their linear actuation. SEM images of PPy composites were performed to determine if any change in morphology took place if EG as solvent in electropolymerization was applied. EDX spectroscopy of PPyPT, PPyPT-EG, PPyCDC and PPyCDC-EG were conducted to evaluate which elements can be detected after actuation cycles in an oxidized and reduced state. Chronopotentiometric measurements of PPy composite films were performed to determine the specific capacitance and their potential application in energy storage related material.

## 2. Materials and methods

### 2.1. Materials

Carbide derived carbon (CDC, amorphous TiC-800, specific surface area of 1470 m^2^ g^−1^, total volume of pores 0.71 cm^3^ g^−1^, volume of micro-pores 0.59 cm^3^ g^−1^ and average pore size of 0.97 nm) was obtained from Skeleton Technologies Ltd. (Tallinn, Estonia). Sodium dodecylbenzenesulfonate (NaDBS, 99%), ethylene glycol (EG, 99.8%), bis(trifluoromethane) sulfonimide lithium salt (LiTFSI, 99.95%), phosphotungstic acid hydrate (PTA, PW_12_O_40_^3−^, reagent grade), ethanol (technical grade) and propylene carbonate (PC, 99%) were obtained from Sigma-Aldrich (Taufkirchen, Germany) and used as supplied. Pyrrole (Py, ≥98%) from Sigma-Aldrich was distilled prior to use and stored at low temperature. Milli-Q+ (deionized water, Tallinn, Estonia) was applied as a solvent. 

### 2.2. Electropolymerization 

Electrochemical synthesis of PPyDBS-PT (PPy-PT) and PPyDBS-PT-CDC (PPyCDC) films was carried out in a two electrode electrochemical cell. PPyPT films were electropolymerized containing 0.01 M phosphotungstic acid, 0.1 M NaDBS in and 0.1 M pyrrole. PPyCDC films were made in the same way with the addition of 1 wt.% of CDC using 0.1 M PTA to solubilise CDC particles in a monomer solution. As a solvent EG:Milli-Q+ solution with ratio 1:1 was applied for PPyCDC and PPyPT composites. In the case of PPyPT-EG and PPyCDC-EG samples, pure EG was applied as the solvent in electropolymerization. Both solutions were ultrasonically dispersed (Hielscher UP200S, 200W, 24 kHz, Mount Holly, NJ, USA) for 30 min in an ice bath and stored in the fridge.

Galvanostatic polymerization was carried out at 1.8 mA for 11.1 h at −20 °C temperature, using a stainless steel sheet (18 cm^2^) as a working electrode and a stainless steel net as a counter and reference electrode. After polymerisation the composite films were washed in ethanol to remove residues of pyrrole and in water to remove excess of NaDBS and PTA. The composite films were stored in 0.1 M NaDBS aqueous solution. The film thicknesses of the composites after drying in an oven at 60 °C (2 mbar, 12 h) were determined over an electronic micrometre gauge meter (Dainu, 0.001 mm sensitivity) and found in a range of 21 ± 2 µm with a weight of 250 ± 30 μg. Additionally, pristine PPy/DBS was made in similar galvanostatic electropolymerization using 0.1 M NaDBS and 0.1 M Pyrrole in aqueous solution either with EG:Milli-Q+ (1:1) and pure EG solvent, resulting in linear films with thickness in the range of 19 ± 2 μm. The PPy/DBS films were made for analytical characterizations to compare those with PPy composite samples.

### 2.3. Linear Actuation Measurements

The film samples (PPyPT, PPyPT-EG, PPyCDC and PPyCDC-EG) were cut in lengths of 1.0 cm and widths of 0.1 cm, fixed on a lower arm that contained gold contacts connected in stretched position to the upper clamp with the force sensor (TRI202PAD, Panlab, Barcelona, Spain). The three electrode cell contained the electrolyte (0.2 M NaClO_4_ in propylene carbonate or aqueous solvents) with the lower clamp set as the working electrode with a platinum sheet counter electrode and an Ag/AgCl (3M KCl) reference electrode. The change in length (constant force of 9.8 mN, length between both clamps was set to 4 mm) was determined from the linear muscle analyser [22] connected over the in-house software with the potentiostat (Biologic PG581, Seyssinet-Pariset, France). Prior to measurement the film samples were stretched up to 1% in the electrolyte for 12 h. Cyclic voltammetry (scan rate 5 mVs^−1^) and square potential steps (frequencies 0.0025 to 0.1 Hz) at a potential range of 0.65 to −0.6 V were carried out for all applied composite samples. The stiffness (mg/μm) of the composites were determined before strain measurements, giving values (Young’s modulus) of before and after actuation shown in Table S1. To determine the diffusion coefficients at oxidation/reduction the Equations (3) and (4) were applied [23].

(3) $$ln\left[1-\frac{Q}{Q_{t}}\right]=-b\cdot t$$

(4) $$D=\frac{b\cdot h^{2}}{2}$$

The charge density *Q* at each time *t* obtained from current density time curves at oxidation/reduction was divided through the total charge density *Q_t_*. The expression of Equation (3) was plotted against time *t* and the slope *b* was determined [24]. With the thickness h of the film samples the diffusion coefficients at oxidation and reduction (Equation (4)) were calculated. Chronopotentiometric measurements at frequencies 0.0025 to 0.1 Hz at applied current densities *j* of ±0.05, ±0.1, ±0.2, ±0.5, ±1 and ±2 A g^−1^ were applied at constant charge densities ±10 C g^−1^. The specific capacitance *C_s_* of the film samples was calculated regarding Equation (5) and obtained from the slope at discharging potential time curves (after the IR drop).

(5) $$C_{s}=\frac{j}{-slope}$$

### 2.4. Characterization

Scanning electron microscopy images of surface and cross sections of dried composite samples after polymerization were made (SEM, 8 kV, Helios NanoLab 600, FEI, Hillsboro, OR, USA). The ion content of the samples was determined with EDX spectroscopy (EDX with X-Max 50 mm^2^ detector, Oxford Instruments, High Wycombe, UK), made at oxidized (5 min at 0.65 V) and reduced states (5 min at −0.6 V) after actuation cycles. Raman spectroscopy using a Renishaw (Wotton-Under-Edge, UK) via micro-Raman spectrometer (514 nm argon-ion laser) was applied to pristine PPy composites. CDC material (TiC-800) was measured in powder formation (KBr pellet) for Raman spectroscopy, and Fourier-transform infrared (FTIR) spectroscopy (Bruker Alpha spectrometer using a Platinum ATR, Billerica, MA, USA) of the PPy samples was conducted. The electronic conductivity of the film samples in oxidized state either direct after polymerization or after actuation cycles (washed with ethanol and Milli-Q+, then dried for 12 h at 2 mbar in an oven) was determined over the four-point-probe conductivity meter (Jandle 4-Point Probe Head, Model RM2, Leighton Buzzard, UK).

## 3. Results and Discussions

### 3.1. Electropolymerization, SEM, EDX and Electronic Conductivities

**Figure 1:**
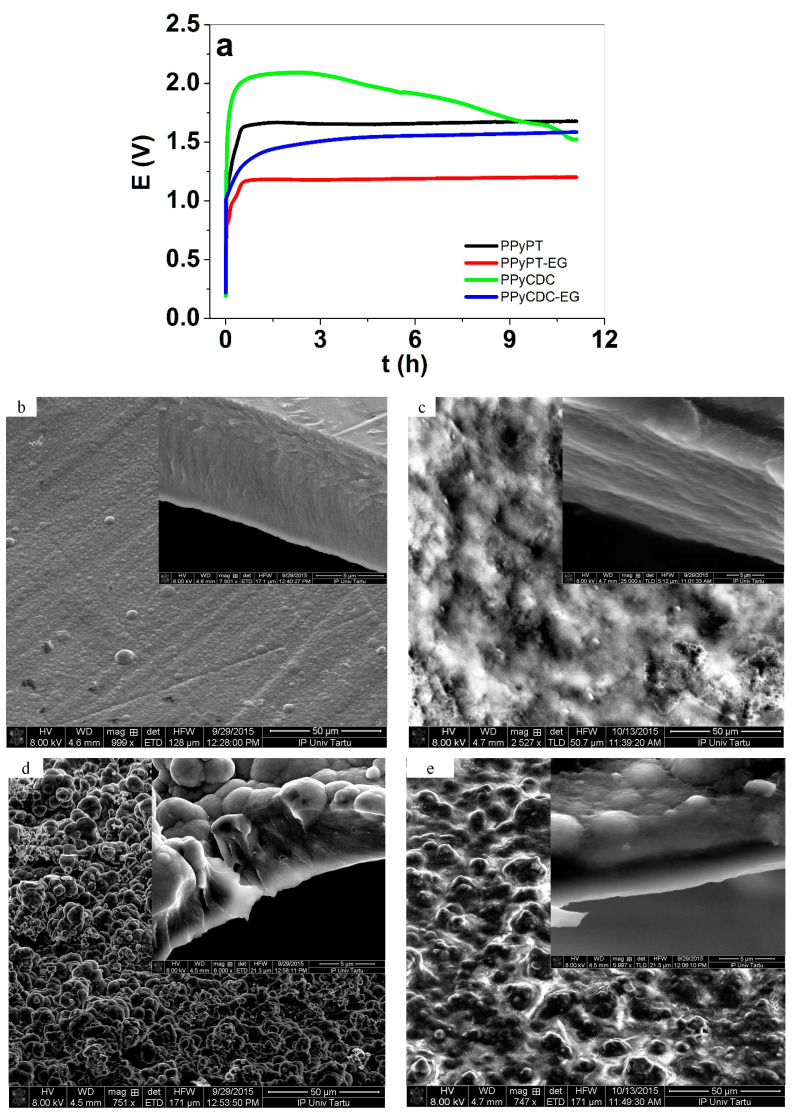
(**a**), Electropolymerization curves are presented of PPyPT (black), PPyPT-EG (red), PPyCDC (green) and PPyCDC-EG (blue). The SEM surface images (scale bar 50 μm) with insets of cross sections (scale bar 5 μm) are shown in (**b**), of PPyPT, (**c**), PPyPT-EG, (**d**), PPyCDC and (**e**), PPyCDC-EG.

Previous research [25] revealed that addition of PTA, well known as a catalyst and mediator [26] in galvanostatic polymerization, led to potential reduction. The electropolymerization curves of composite samples are presented in Figure 1a and the SEM images with insets of cross-sections are shown in Figure 1b–e.

The electropolymerization curves shown in Figure 1a reveal for composite samples polymerized in EG solvent such as PPyPT-EG and PPyCDC-EG a reduced potential in comparison to those polymerized in EG Milli-Q+ mixture. In general, if the potential is reduced in electropolymerization the resulting films shown from previous research [27] become more compact and dense. In the case of PPyCDC (Figure 1a) the decrease of the potential time curve is much more pronounced in comparison to other samples. The main reason for this behavior relates to the CDC-PT particles embedded mostly at the surface, leading to increasing porosity and roughness of the PPyCDC film. A similar tendency was observed of PPyCDC without PTA in former research [28]. The SEM image of PPyPT had a smooth surface with a relatively compact cross section (Figure 1b with inset) while the PPyPT-EG samples revealed a different morphology of probably EG units on the PPy surface with the cross section image shown as well in dense morphology (Figure 1c with inset). The inclusion of CDC in electropolymerization with the addition of PTA shown for PPyCDC (Figure 1d) had a quite rough surface morphology with CDC particles on the surface being more apparent in the cross section image (Figure 1d inset). Surprisingly, the PPyCDC-EG films reveal a different morphology, with CDC particles embedded in the PPy structure seen as well in cross section images in Figure 1e with more compact and dense film. The electronic conductivities of dry samples directly after electropolymerization as well those after actuation cycles in the oxidized state (0.65 V) in aqueous and organic electrolytes are presented in Table 1.

The PPyPT and PPyCDC films showed best conductivities in the pristine state while PPyPT-EG and PPyCDC-EG films are 5 times reduced (Table 1). The electronic conductivity for all samples after actuation was found lower in NaClO_4_-PC, which was shown from previous research [12] that the nature of the solvent has a strong influence on the electronic conductivity on PPy films. In aqueous electrolyte (NaClO_4_-aq, Table 1) the conductivity increased 1.3 times for PPyPT and PPyCDC. Interestingly, a 4 times higher conductivity was found for PPyPT-EG and PPyCDC-EG in aqueous electrolyte. Previous research [18] using PPy-DBS-PEG composites revealed that the conductivity with PEG was increased, which was related to the morphology of the samples as well having less cross-linkage and defects. In general, the trend of PPy composites (Table 1) is that in aqueous NaClO_4_ electrolyte the electronic conductivity improves. 

**Figure 2:**
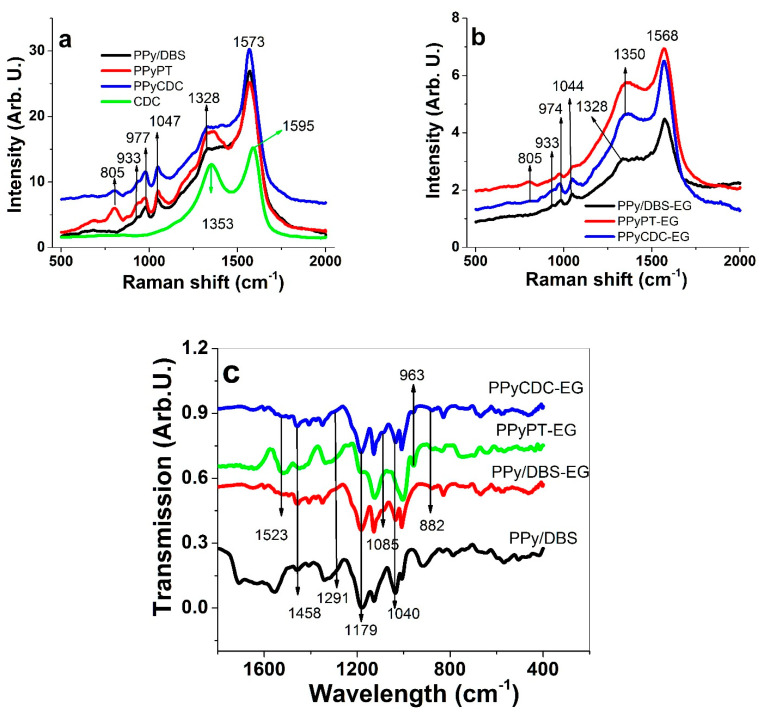
Raman spectroscopy (500–2000 cm^−1^, 514 nm) of PPy composites (PPy/DBS, black line; PPyPT, red line; PPyCDC, blue line) presenting in (**a**), PPy composites made in EG:Milli-Q+ mixture with addition of CDC (green line) and in (**b**), those made in pure EG solvent. Presented in (**c**), FTIR spectroscopy (400–1800 cm^−1^ with PPy/DBS (black line), PPy/DBS-EG (red line), PPyPT-EG (green line) and PPyCDC-EG (blue line).

Raman and FTIR spectroscopy of PPy composites included pristine PPy/DBS and CDC are performed with results shown in Figure 2a–c. 

Typical PPy signals of PPy composites (Figure 2a) are found at 933 cm^−1^ (radical cations, due to the absence of the 1083 cm^−1^ peak [29]). The double peaks at 977 cm^−1^ (C-H bending [30] with evidence to cationic behavior [31]) and 1047 cm^−1^ (in plane symmetric bending [30]) are associated to the polaron structure [32]. The ratio between those double peaks were 0.83 for PPy/DBS, 0.92 for PPyPT and 0.93 for PPyCDC, which indicates higher relative polaron (radical cation) content in PPyPT and PPyCDC. The small peaks at 1328 cm^−1^ belong to the C-C stretching [30]. The dominant peak in Figure 2a at 1573 cm^−1^ refers to C=C stretching [30]. The PTA typical signals in Raman spectroscopy can be found between 904 to 1007 cm^−1^ [33], which could not be detected in the PPyPT or PPyCDC due to overlapping peaks of PPy. A strong peak at 805 cm^−1^ for PPyPT and in lower intensity for PPyCDC belong to tungsten trioxide (WO_3_) [33], confirming that PTA was successfully incorporated. From the Raman spectrum of CDC only two peaks can be identified, disorder-induced D peak at 1353 cm^−1^ and the graphite G-peak at 1595 cm^−1^ [34]. Figure S1 shows the narrow area of Raman shifts at 1350 to 1450 cm^−1^ identifying a small peak at 1353 cm^−1^ of the D peak of CDC in PPyCDC composites.

Comparing Figure 2a with the PPy composites made in EG solvent (Figure 2b) there are certain differences in some peaks while others such as the tungsten trioxide WO_3_ peak at 805 cm^−1^ and the 933 cm^−1^ peak identified as radical cations are found at the same position. The strong peak of C=C stretching is shifted to 1568 cm^−1^, revealing that lower doping level and therefore less conductive PPy composites [35] are obtained. The 1328 cm^−1^ peak (C-C stretching) was detected only in PPy/DBS-EG, while PPyPT and PPyCDC showed another broad peak at 1350 cm^−1^ that belongs to the ring stretching mode of the polymer backbone [36] overlapping the CDC signals of the D peak (Figure 2a). The double peaks of the C-H in plane deformation (oxidized level of PPy [35]) did shift as well to 974 and 1044 cm^−1^ with ratios found for PPy/DBS-EG at 0.63, PPyPT-EG at 0.8 and PPyCDC-EG at 0.87 having much lower polaron content in comparison to PPyDBS, PPyPT and PPyCDC samples. The polaron content is related to the conductivity [37], agreeing well with the 5 times lower conductivity of PPy composites made in EG (Table 1). The EG peaks were not identified in Raman due to strong PPy peaks overlapping those peaks. 

The FTIR spectra (Figure 2c) reveal typical PPy ring vibrations [38] located at 1545 cm^−1^ shown only for pristine PPy/DBS with 1523 cm^−1^ identified as N-H bending [25] seen in PPy/DBS-EG, PPyPT-EG and PPyCDC-EG. The 1458 cm^−1^ peaks shown in all PPy composites belong to PPy ring vibrations [38] with additional peaks seen at 1291 cm^−1^, 1040 cm^−1^ (C-H in plane vibrations) and the C-N stretching vibration was located at 1175 cm^−1^. The PTA peaks at 963 cm^−1^ (W-O stretching vibration) are shown only for PPyPT-EG and PPyCDC-EG composites. PPy composites made in EG have two peaks that identify EG integration [39] in PPy with one at 1085 cm^−1^ (C-O stretching) and one at 882 cm^−1^ that was identified as CH_2_ rocking vibration.

In summary, the characterization given by Raman spectroscopy revealed identification of compounds in PPy composites such as PTA and CDC as well the PPy composites made in EG having lower conductivities after formation shown in shifts of peaks in Raman spectroscopy. FTIR spectroscopy could identify PTA inclusion as well as all PPy signals shown with additional EG peaks revealed.

**Figure 3:**
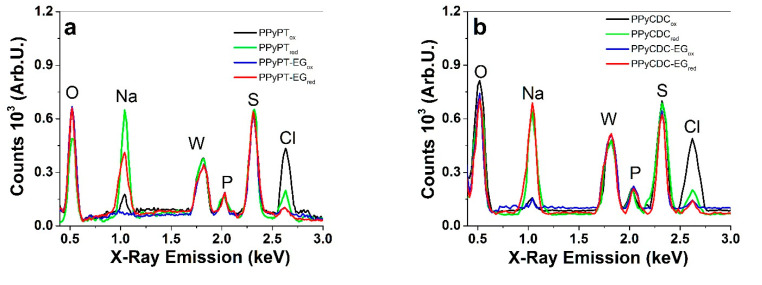

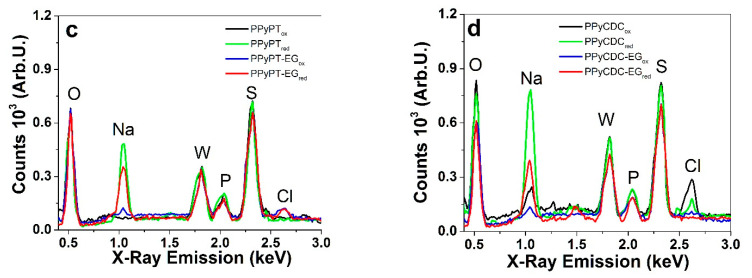
(**a**), EDX spectroscopy of cross-section images of PPy samples polymerized in EG:Milli-Q+ at oxidation such as PPyPT_ox_ and PPyCDC_ox_ (black line) and at reduction PPyPT_red_ and PPyCDC_red_ (green line) as well those PPy films polymerized in EG at oxidation with PPyPT-EG_ox_ and PPyCDC-EG_ox_ (blue line) and at reduction PPyPT-EG_red_ and PPyCDC-EG_red_ (red line), and in (**b**) PPy samples in NaClO_4_-PC and in (**c**,**d**), those in NaClO_4_-aq electrolytes.

To investigate the ion-contents of oxidized and reduced PPy samples EDX spectroscopy of cross-section images was performed and the results are shown in Figure 3a–d. EDX spectra of pristine PPy composites including PPy/DBS directly after polymerization (in oxidized state, ~0.6 V) are presented in Figure S2a,b. The spectra with included carbon peaks are shown in Figure S3a–d. 

From Figure S3a–d the carbon peak (C) is shown at 0.26 keV and found slightly increased with the addition of CDC materials (Figure S3b,d). In Figure 3a–d the oxygen peak (O) shown at 0.52 keV refers to PTA (PW_12_O_40_^3^^−^) as well DBS^−^ molecules immobilized in PPy and partly from the applied electrolyte NaClO_4_. The sodium peak (Na) is shown at 1.04 keV, referring to the cation Na^+^ incorporated mainly at reduction. The tungsten peak (W) at 1.78 keV and the phosphor peak (P) at 2.02 keV represent the incorporated PT^4^^−^ in the PPy network and the sulphur peak (S) characterized the immobile DBS^−^ anion. The chloride peak (Cl) found at 2.62 keV belongs to the anions ClO_4_^−^. The PPyPT and PPyCDC films in NaClO_4_-PC electrolytes in Figure 3a,b revealed that at oxidation a chloride peak (ClO_4_^−^ anions) and at reduction a sodium peak (Na^+^ cations) were detected, hinting of a mixed ion process. From previous research [40] the change of solvent of aqueous to propylene carbonate in pristine PPy/DBS showed that the anion-driven process was obtained due to a special phenomenon that the immobile DBS^−^Na^+^ cannot dissociate in the propylene carbonate solvent. Therefore Equation (2) describes the reaction that the expected cation-driven actuator becomes anion-driven. Figure 3a revealed for PPyPT at reduction a chloride peak, which we assume relates to ClO_4_^−^ anions remaining in the PPyPT network and leading to Na^+^ anions ingress to compensate for the negative charges. Similar behavior was revealed for other conducting polymers such as PEDOT or PPy/CF_3_SO_3_, where the triflate (CF_3_SO_3_^−^) anions found immobile led to mixed ion actuation at redox cycles [41]. 

In the case of PPyPT-EG and PPyCDC-EG samples a strong sodium peak is shown in reduction, which led to the conclusion that the cations Na^+^ are moving in, and at oxidation out (no Na peak at oxidation detected), considering the cation-driven actuation mechanism seen from Equation (1). In case of aqueous NaClO_4_ electrolyte shown in Figure 3c,d, the sodium peak is found dominant at reduction for PPyPT and PPyCDC as well to analogous PPyPT-EG and PPyCDC-EG samples. In case of PPyCDC at oxidation and reduction (Figure 3d) sodium and chloride peaks are found in small parts, which we assume is the reason for the incorporated meso-porous CDC particles, whereas ions can be injected surrounded by counterions forming an electrical double layer [42]. PPyCDC-EG (Figure 3d) revealed main sodium incorporation at reduction to compensate for the negative charge of the immobile DBS^−^ and PT^4^^−^ that surround the CDC particles. The pristine PPy/DBS samples (made in EG:Milli-Q+ and in EG solvent) shown in Figure S2a,b revealed that DBS^−^ anions are immobile referring to oxygen peak and sulphur signals. For PPyPT (PPyPt-EG) and PPyCDC (PPyCDC-EG) additional tungsten and phosphor peaks are shown, as well as for those made in EG solvent. In the case of PPyCDC (PPyCDC-EG) a sodium peak was detected which we assume is incorporated in the meso-porous CDC material located on the near surface of the composite films. 

### 3.2. Linear Actuation

The linear actuation properties of the PPy samples in NaClO_4_-PC as well NaClO_4_-aq electrolytes were performed with included cyclic voltammetry and square potential steps. PPy doped with DBS^−^ anions revealed from previous research their actuation direction changed in organic electrolytes due to non-dissociated cation-DBS molecules inside the PPy network; therefore mainly expansion at oxidation [40] was discovered, shown as well for PPyCDC samples [13]. The influence of EG in polymerization without addition of Milli-Q+ is studied in this work, compared to those made in EG:Milli-Q+ 1:1.

#### 3.2.1. Cyclic Voltammetry

**Figure 4:**
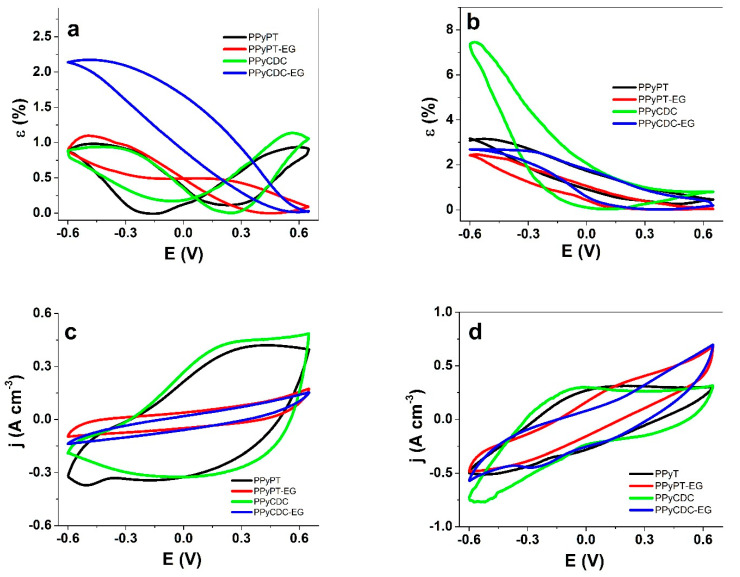
Cyclic voltammetry (scan rate 5 mV s^−1^) of PPyPT (black line), PPyPT-EG (red line), PPyCDC (green line) and PPyCDC-EG (blue line) at applied potential range 0.65 to −0.6 V, showing strain ε against potential E of (**a**), in NaClO_4_-PC and in (**b**), NaClO_4_-aq electrolyte, in (**c**), the current density potential curves of PPy composites in NaClO_4_-PC and (**d**), in NaClO_4_-aq.

Cyclic voltammetry (scan rate 5 mV s^−1^) with linear actuation measurements in strain are shown for composite films (PPyPT, PPyPT-EG, PPyCDC, PPyCDC-EG) in NaClO_4_-PC electrolyte in Figure 4a with current density potential curves presented in Figure 4c. The strain values in NaClO_4_-aq electrolyte are compared in Figure 4b and the current densities potential curves are shown in Figure 4d. The corresponding charge densities are presented in Figure 4a,b.

Figure 4a reveals for PPyPT and PPyCDC mixed linear actuation with nearly equal expansion at oxidation/reduction in the range of 1% strain. Surprisingly, PPyPT-EG and PPyCDC-EG had main expansion at reduction with 1% strain for PPyPT-EG and 2.1% strain for PPyCDC-EG, shown as well in Figure 3a,d in EDX measurements that show main ingress of Na^+^ cations taking place for those composites. From previous research PPy/DBS trilayer [12] or PPyPT and PPyCDC linear films [13] revealed main expansion at oxidation explained with the effect of PC solvent. One possible explanation shown from former research [21] using instead of EG polyethylene glycol (PEG) showed that in aqueous electrolyte the affinity to metallic cations was enhanced due to PEG chains forming complexes with cations. 

The current density potential curves of PPyPt-EG and PPyCDC-EG (Figure 4c) revealed nearly 4 times lower current density, which is reflected in the much lower electronic conductivity (Table 1) in comparison to PPyPT and PPyCDC. It was found from previous research [43] that the charge transport in conducting polymers was enhanced in highly organized islands with high conductivity. Another factor is the difference in the ion mobility; that can be the reason for the mismatch between exchanged charge density as well as conductivities. In the case of PPyPT-EG and PPyCDC-EG lower current density and charge density might be, besides the lower electronic conductivity, also the mobility of ions finding a barrier in propylene carbonate that leads to the lower charge densities. In the case of PPy film samples polymerized in EG the current density curves did not reveal any oxidation/reduction peak while PPyPT had an oxidation wave at 0.42 V and a reduction wave at −0.37 V. Pristine PPy/DBS in aqueous electrolyte had a reduction peak in range of −0.5 V [44], whereas the shift in the reduction wave regarding PPyPT can be explained with the nature of POM (polyoxometalates) molecules having antioxidant properties [45]. PPyCDC showed an oxidation wave at 0.22 V with no reduction waves. The charge density potential curves shown in Figure S2a reveals 4.4 times higher charge densities (~60 to 65 C cm^−3^) for PPyPT and PPyCDC in comparison to those made in EG (~16 C cm^−3^). 

In the case of aqueous NaClO_4_ electrolyte on PPy composites (Figure 2b), for all applied film samples having main expansion at reduction with a high strain of 7.7% for PPyCDC films as well showed minor expansion at oxidation in range of 0.8%, while all other samples (PPyPT, PPyPT-EG and PPyCDC-EG) found in a similar range of 2.3–3.2% strain. The current density potential curves shown in Figure 4d were similar for all applied PPy samples, displayed as well from the charge density curves in Figure S4b, where those polymerized in EG revealed charge densities in the range of 33 to 35 C cm^−3^ and those polymerized in EG:Milli-Q+ had a range of 40 to 44 C cm^−3^. The PPyPT films (Figure 4d) showed an oxidation wave at 0.03 V and a reduction wave at −0.42 V, similar to those from previous research [25], while for PPyCDC the oxidation wave was shifted to more negative values with −0.16 V with a reduction wave at −0.52 V [27]. 

In summary, PPy composites made in EG have low current and charge densities in NaClO_4_-PC electrolyte, revealing main expansion at reduction while PPyPT and PPyCDC samples had mixed ion actuations. In NaClO4-aq electrolyte all investigated PPy films had expansion at reduction with maximum strain found for PPyCDC films. Further analysis of linear actuation applied in square potential steps will give more information how the strain vs. charge density influences the actuation properties of PPy composite samples.

#### 3.2.2. Square Potential Steps

**Figure 5:**
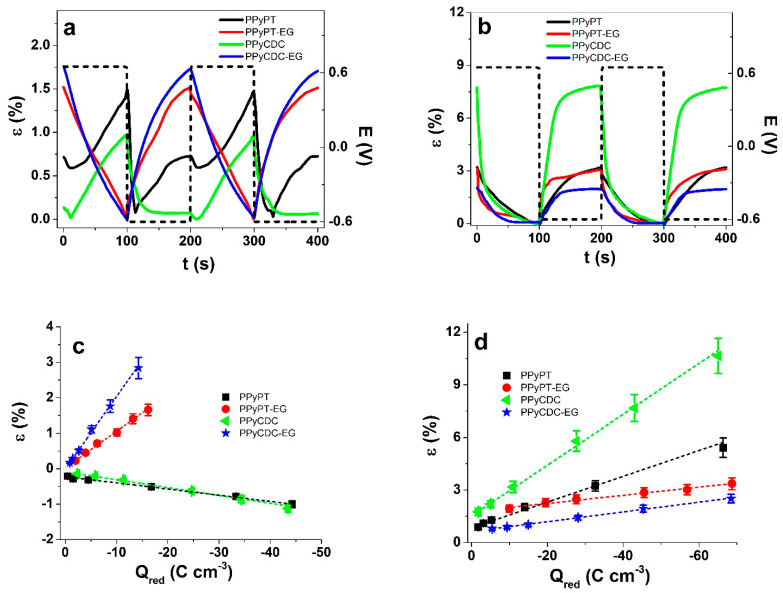
Square potential step measurements of PPyPT (black line, ■), PPyPT-EG (red line, ●), PPyCDC (green line, ◄) and PPyCDC-EG (blue line, ★) showing the strain ε at frequency 0.005 Hz of two subsequent cycles (2nd to 3rd) in (**a**), NaClO_4_-PC electrolyte and in (**b**), NaClO_4_-aq electrolyte at applied potential range E (dashed line), in (**c**), the strain ε against charge density Q_red_ at reduction in NaClO_4_-PC are presented and in (**d**), in NaClO_4_-aq are shown The dashed lines in c and d represent the linear fit and are shown here for orientation, only.

The strain against time of two subsequent cycles at applied frequency 0.005 Hz of PPyPT, PPyPT-EG, PPyCDC and PPyCDC-EG samples are shown for electrolyte NaClO_4_-PC in Figure 5a and for NaClO_4_-aq in Figure 5b. The current density time curves for those two subsequent cycles are presented in Figure S5a,b, respectively. The strain against applied frequencies 0.0025 to 0.1 Hz in both solvents are shown in Figure S5c,d. Negative strain represents expansion at oxidation and positive strain refers to expansion at reduction. From each current density time curve at each applied frequency the charge density was determined over integration. The results of strain against charge density at reduction are shown for PPy composite samples in electrolyte NaClO_4_-PC in Figure 5c and for NaClO_4_-aq in Figure 5d.

The strain against time shown in Figure 5a in NaClO_4_-PC electrolyte reveals for PPyPT composite films mixed actuation with main expansion at oxidation. Taking into account that incorporation of PTA in the PPyDBS network led to an increase of negative charges (PT^4−^) with incorporation of cations at reduction reflecting the mixed ion-actuation, shown as well from previous research [13]. In the case of PPyCDC shown in Figure 5a only a small expansion at reduction was obtained with the main expansion at oxidation. Expansion at oxidation due to solvent PC was shown previously on PPyDBS linear actuators [40]. In the case of PPyCDC composite samples we have two different mechanisms, one is that the CDC particles follow the non-faradaic process [46] and PPy follows the faradaic process [47]. In comparison to those samples made in EG such as PPyPT-EG and PPyCDC-EG only expansion at a reduction in the range of 1.6% was found. The inclusion of EG is revealed as well in SEM images having a less porous and smoother surface (Figure 1c,e). As a consequence, we assume that the incorporation of PC molecules in PPy composites made in EG are reduced, leading to expansion at reduction by incorporation of Na^+^ cations.

If those samples were investigated in aqueous NaClO_4_ electrolyte (Figure 5b) main expansion at reduction was found for PPyPT and PPyPT-EG in the range of 3.3% strain whereas PPyCDC revealed a best strain of 8%. PPyCDC-EG expansion at a reduction was found lowest in this study, with 2% strain. Previous research [27] on PPyCDC applied in aqueous LiTFSI electrolyte revealed a strong increase of strain due to the decrease of Young’s modulus nearly 6 times if CDC particles incorporated, shown here as well in the case of PPyCDC in nearly 4 times lower modulus than PPyCDC-EG (Table S1). If comparing the surface morphology of PPyCDC and PPyCDC-EG (Figure 1c,e), the CDC particles can be seen clearly on surface, while in PPyCDC-EG the CDC particles included in the PPy network have a less porous morphology, which we assume is the main reason for a similar Young’s modulus before and after actuation, shown in Table S1. 

**Figure 6:**
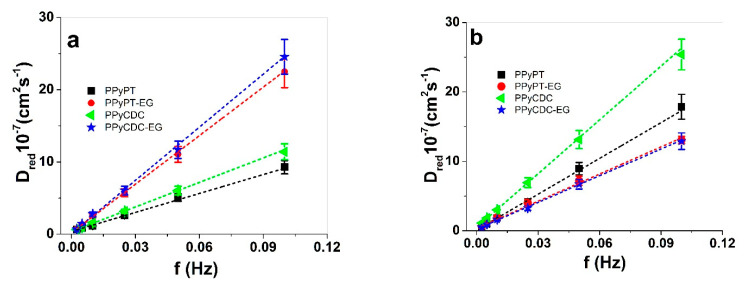
Diffusion coefficients at reduction D_red_ calculated for PPyPT (■), PPyPT-EG (●), PPyCDC (◄) and PPyCDC-EG (★) showing in (**a**), in NaClO_4_-PC and (**b**), NaClO_4_-aq electrolyte against applied frequencies f (0.0025 Hz to 0.1 Hz). The dashed lines represent the linear fit and are shown only for orientation.

The strain against charge densities at reduction for PPy samples (Figure 5c,d) revealed in both electrolytes that the strain increased nearly linearly with increasing charge densities referring to faradaic process [47], following the ESCR model [48]. PPyPT and PPyCDC in NaClO_4_-PC electrolyte presented in Figure 5c had a negative strain (expansion at oxidation) within similar range (0.0025 Hz, Figure S5c) of −1 ± 0.1% (charge densities −43.5 ± 4.1 C cm^−3^). The PPyPT-EG (strain of 1.7 ± 0.15%) and PPyCDC-EG (2.9 ± 2.6%) revealed expansion at reduction with 3 times lower charge densities in comparison to PPyPT and PPyCDC composite samples. The strain against charge densities at reduction presented in NaClO_4_-aq electrolyte (Figure 5d) revealed for all PPy composite samples expansion at reduction with high strain found for PPyCDC in the range of 10.6 ± 1.1% (frequency 0.0025 Hz, Figure S5d). The main reason that the strain of PPyCDC was so different from other samples is the decrease of Young’s modulus shown in Table S1. For PPyPT, PPyPT-EG and PPyCDC-EG the modulus decreased only in small numbers before and after actuation. The charge densities for all applied PPy composites were found nearly equal with −67 ± 6.3 C cm^−3^ at applied frequency 0.0025 Hz, revealing that in aqueous electrolyte other factors were influencing the strain than the charging/discharging properties. To investigate the diffusion coefficients at reduction Equations (3) and (4) was applied for PPy composite samples and the results in electrolyte NaClO_4_-PC and NaClO4-aq (diffusion coefficients at oxidation are shown in Figure S6a,b) are presented in Figure 6a,b, respectively.

Figure 6a,b reveals a general trend that with increasing frequency the diffusion coefficients at reduction increased as well (shown as well for the diffusion coefficient at oxidation in Figure S6a,b). The reason for this relied on different kinetic processes taking place on PPy composites while low diffusion coefficients at low frequencies providing longer time leading to shrinking, compaction, relaxation, and swelling process following the ESCR model [48]. At shorter times (higher frequency) only shrinking and swelling took place. The diffusion coefficients at reduction in NaClO_4_-PC electrolyte (those at oxidation D_ox_ shown in Figure S6a) revealed that for PPyPT-EG and PPyCDC-EG nearly 2 times higher diffusion coefficients at reduction (oxidation) were found in comparison to PPyPT and PPyCDC. From linear actuation cycles (Figure 5a) the PPyPT-EG and PPyCDC-EG had expansion at reduction showing cation-driven actuation. In the case of PPyPT and PPyCDC mixed ion actuation (Figure 4a and Figure 5a) was observed that influenced the diffusion coefficients; they were 1.2 times reduced in (Figure 6a) comparison to PPy composites made in EG. 

Figure 6b represents the PPy composite films in NaClO_4_-aq electrolyte with diffusion coefficients at reduction (Figure S6b diffusion coefficients at oxidation) that revealed highest diffusion coefficients for PPyCDC composites followed by PPyPT while PPyPT-EG and PPyCDC-EG were found in a similar range. The linear actuation response of PPyCDC (Figure 4b and Figure 5b) had the best strain in this study with main expansion at reduction; as mentioned before the CDC particles seen on the surface of PPyCDC samples led to more porous morphology (Figure 1d) that we assume enhanced the ion mobility (higher diffusion coefficients). In the case of PPyCDC-EG the more compact and less porous morphology with CDC embedded in the PPy network (Figure 1e) reduced the diffusion coefficient and was found to be 2.4 times lower.

In summary, the nature of the applied EG solvent in polymerization regarding PPyPT-EG and PPyCDC-EG reveals only one actuation direction independent of which solvent (aq or PC) is applied, making such materials more reliable as an actuator. In the case of PPyPT and PPyCDC polymerized in EG:Milli-Q+ mixture the mixed actuation in NaClO_4_-PC are not favorable for application to achieve high strain. Higher strain can be achieved for PPyCDC in NaClO_4_-aq electrolyte up to 10%.

### 3.3. Energy Storage

**Figure 7:**
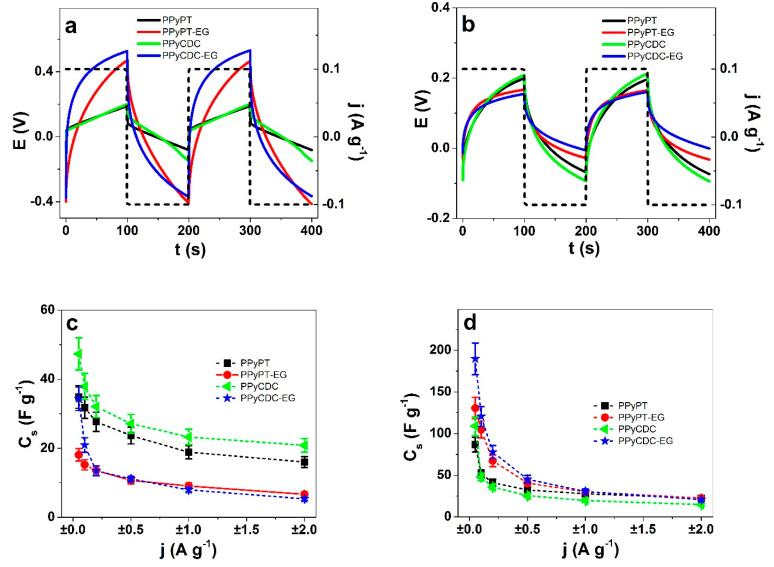
Chronopotentiometric measurements of PPy samples at applied current densities ±0.05 to ±2 A g^−1^ having same charge density of ±10 C g^−1^ showing in the potential time curves (2nd and 3rd cycles) at current density ±0.1 A g^−1^ (dashed line) of PPyPT (black curve), PPyPT-EG (red curve), PPyCDC (green curve) and PPyCDC-EG (blue curve) in (**a**), NaClO_4_-PC and (**b**), NaClO_4_-aq electrolytes. Presented in (**c**), the specific capacitance *C_s_* (calculated over Equation (3)) of PPyPT (■), PPyPT-EG (●), PPyCDC (◄) and PPCDC-EG (★) against applied current densities *j* in NaClO_4_-PC and (**d**), NaClO_4_-aq electrolytes.

The need for soft materials, bendable and applicable in supercapacitor material are in recent times a focus for high capacitive electrodes. CDC material is well known for its excellent energy storage properties [49] due to its meso-porous nature. It is therefore of interest to investigate the energy storage capability on PPy composites (PPyPT, PPyPT-EG, PPyCDC and PPyCDC-EG) by determining their specific capacitance (Equation (5)) obtained over chronopotentiometric measurements. The potential time curves of PPy composites in NaClO_4_-PC are presented in Figure 7a and in NaClO_4_-aq in Figure 7b. The specific capacitance against applied current densities ±0.05 to ±2 A g^−1^ in NaClO_4_-PC are shown in Figure 7c and those in NaClO_4_-aq in Figure 7d. From each applied PPy composite at least three samples are made and the results are shown in mean values with standard deviations.

Stating from the ESCR model if the potential time curves of two subsequent cycles are congruent in the applied potential range 0.65 to −0.6 V, then the charging/discharging is in balance [50] and avoids the over-reduction and over-oxidation processes, shown for all applied PPy samples. The potential time curves of the chronopotentiogram in NaClO_4_-PC electrolyte for PPyPT-EG and PPyCDC-EG in Figure 7a showed 3.2 times higher potentials than PPyPT and PPyCDC. The main reason relies on the higher resistivity for PPyPT-EG and PPyCDC-EG (reflecting in lower electronic conductivity, Table 1). In the case of NaClO_4_-aq electrolyte shown in Figure 7b the potential time curve is nearly in the same range with 1.3 times lower profiles shown for PPyPT-EG and PPyCDC-EG films. From Equation (5) the slope at discharging (after IR drop) was taken and with current density *j* the specific capacitance *C_s_* was calculated. Conducting polymers such as PPy applied in this work are pseudo-capacitors [51] in their charging/discharging characteristics following faradaic processes. CDC particles are included in PPy, following non-faradaic processes [42]. The disadvantage of pristine electrochemically prepared PPy having a dense growth with therefore high density material obtained, was a reduction of the capacitance per gram [52]. The main reason for such relies on accessibility of ions in deeper cavities of the PPy films. Therefore, in most cases PPy composites with carbon materials as well others are applied [53].

In NaClO_4_-PC electrolyte the specific capacitance at 0.05 A g^−1^ for all samples was found relatively low ranging from 47 ± 4 F g^−1^ (PPyCDC) to 34 ± 3.1 F g^−1^ for PPyPT and PPyCDC-EG while the lowest value found with 18 ± 1.7 F g^−1^ for PPyPT-EG. 

Previous research [54] of PPy doped with different types of polyoxometalates in acetonitrile revealed a specific capacitance in range of 25 to 38 F g^−1^, similar to our results. PPyCDC revealed the best specific capacitance in this research with similar values found for CDC particles spray coated on a PVdF membrane [55] in the range of 31–39 F g^−1^ applied in the same solvent PC. Recent research [56] using electropolymerized PPy in combination with polymerized ionic liquids (PIL) forming PPyPIL films led to specific capacitance either in aqueous or organic electrolyte in the range of 75 F g^−1^. 

The specific capacitance in NaClO_4_-aq electrolyte (Figure 7d) at ±0.5 A g^−1^ for PPyCDC-EG had high specific capacitance in the range of 190 ± 18 F g^−1^ followed with PPyPT-EG with the range of 130 ± 14 F g^−1^. Therefore, PPy composite made in EG revealed the best specific capacitance in this study, showing its utilization in energy storage application. PPyCDC specific capacitance was found in the range of 109 ± 11 F g^−1^ and PPyPT had a range of 87 ± 8 F g^−1^ at ±0.05 A g^−1^ current density (Figure 7d). Previous research also compared different PTA concentrations whereas the lowest concentration of 0.005 M of PPyPT films had the best specific capacitance of 223 F g^−1^ (±0.09 A g^−1^) [25]. Other research found that PPy with incorporated POM molecules is qualified for energy storage material reaching specific capacitance in the range of 168 F g^−1^ [57]. Pristine PPy/DBS applied in different aqueous electrolytes [10] revealed capacitance between 20 to 60 F g^−1^ (±0.12 A g^−1^). 

Cycle stability of PPy composites are presented in Figure S7a, (NaClO_4_-PC, ±2 A g^−1^, 0.1 Hz) revealing for PPyPT-EG and PPyCDC-EG a decrease of capacitance after 1000 cycles in the range of 60%. PPyPT capacitance decreased nearly 40% while the best cycle stability was found for PPyCDC with retention of capacitance at 80 % after 1000 cycles. A possible explanation why PPyPT-EG and PPyCDC-EG had such high loss of capacitance was shown recently [58]—cation-activity (cation-driven) being the main reason for low cycle stability. Figure S7b revealed for PPy composites in NaClO_4_-aq a better retention of capacitance after 1000 cycles with PPyPT, PPyPT-EG and PPyCDC in the range of at 66–68%. PPyCDC-EG had the best retention of capacitance of 90% (20.6 F g^−1^ at cycle 5 to 18.7 F g^−1^ at cycle 1000). 

Additionally, the inclusion of EG increased the specific capacitance from type PPyCDC-EG that was found 1.7 times more efficient in aqueous electrolyte and the best capacitance retention of 90% in comparison to PPyCDC composites.

## 4. Conclusions

Electropolymerization at low temperature needs anti-freezing agents, for which in general EG is applied mixed with aqueous solvent forming PPy doped with DBS^−^ with the addition of PTA and CDC composite films such as PPyPT and PPyCDC. In this way there is a change of solvent in electropolymerization to pure EG forming PPyPT-EG and PPyCDC-EG composites. Raman and FTIR spectroscopy could identify all additives such as PTA, CDC and EG in PPy composites. Linear actuation of PPy composites regarding their linear actuation response in NaClO_4_-PC and NaClO_4_-aq were compared. PPyPT and PPyCDC revealed mixed ion actuation while those polymerized in EG solvent had only expansion at reduction (1–2% strain) in NaClO_4_-PC. In aqueous NaClO_4_ electrolyte all composite films showed expansion at reduction with the best strain found for PPyCDC in a range of 10% with all others found in the range of 2–3% strain. It is the general goal of conducting polymer actuators having only one expansion direction either at oxidation (anion-driven) or reduction (cation-driven). The novel PPy composites polymerized in EG fulfilled this goal showing strain at reduction (cation-driven) independent of applied solvent with possible applications in soft robotics or smart textiles. The best specific capacitance was found in aqueous electrolyte with 190 F g^−1^ for PPyCDC-EG type as well as best capacitance retention of 90% after 1000 cycles (±2 A g^−1^, 0.1 Hz), making such composite material applicable for flexible energy storage devices.

## Figures and Tables

**Table 1:** Electronic surface conductivities σ_e_ of films of type PPyPT, PPyPT-EG, PPyCDC and PPyCDC-EG in pristine condition (direct after electropolymerization) and after actuation.

PPy Films	Pristine σ_e_ (S cm^−1^)	σ_e_ (S cm^−1^) in NaClO_4_-PC	σ_e_ (S cm^−1^) in NaClO_4_-aq
PPyPT	9.2 ± 0.65	6.3 ± 0.52	11.5 ± 1.06
PPyPT-EG	1.8 ± 0.12	0.7 ± 0.05	7.5 ± 0.64
PPyCDC	7.8 ± 0.68	4.8 ± 0.32	10.1 ± 0.84
PPyCDC-EG	1.5 ± 0.13	0.6 ± 0.04	6.6 ± 0.52

## Data Availability

The data presented in this study are available on request from the Corresponding author.

## Supplementary Materials

The following are available online at https://www.mdpi.com/article/10.3390/ma14216302/s1, Table S1. Young’s modulus Y of samples such as PPyPT, PPyPT-EG, PPyCDC and PPyCDC-EG. Figure S1. Raman spectra’s of pristine PPy films in oxidized state. Figure S2. EDX spectroscopy of pristine PPy/DBS and PPy/DBS-EG, PPyPT and PPyPT-EG and PPyCDC and PPyCDC-EG in oxidized state. Figure S3. EDX spectroscopy at cross section in oxidized state and reduced state of PPy composite samples. Figure S4. Coulovoltammetry of PPyPT, PPyPT-EG, PPyCDC and PPyCDC-EG of charge density against potential E. Figure S5. Square potential steps of of PPyPT, PPyPT-EG, PPyCDC and PPyCDC-EG showing current density time curves. Figure S6. Diffusion coefficients at oxidation Dox of PPyPT, PPyPT-EG, PPyCDC and PPyCDC-E. Figure S7. Cycle stability of the specific capacitance *C_s_* against cycle numbers.
