# Supplementary material for: Polypyrrole with Phosphor Tungsten Acid and Carbide-Derived Carbon: Change of Solvent in Electropolymerization and Linear Actuation
Source: Materials (Basel). 2021 Oct 22;14(21):6302. doi: 10.3390/ma14216302 (PMC8585407; doi:10.3390/ma14216302)
Supplement: Supplementary file 1 [file materials-14-06302-s001.zip › materials-1417294-supplementary.pdf]

materials

MDPI

Supplementary materials

Polypyrrole with Phosphor Tungsten Acid and Carbide-Derived Carbon: Change of Solvent in Electropolymerization and Linear Actuation.

Chau B. Tran [1], Zane Zondaka [2], Quoc Bao Le [3], Bharath Kumar Velmurugan [4] and Rudolf Kiefer [3,*]

[1] Faculty of Applied Sciences, Ton Duc Thang University, Ho Chi Minh City 700000, Vietnam; tranboichau@tdtu.edu.vn
[2] Intelligent Materials and Systems Lab, Faculty of Science and Technology, University of Tartu, Nooruse 1, 50411 Tartu, Estonia; zane.zondaka@ut.ee
[3] Conducting polymers in composites and applications Research Group, Faculty of Applied Sciences, Ton Duc Thang University, Ho Chi Minh City 700000, Vietnam; lequocbao@tdtu.edu.vn (Q.B.L.); rudolf.kiefer@tdtu.edu.vn (R.K.)
[4] Department of Medical Laboratory Science and Biotechnology, Asia University, Taichung 413 Taiwan; bharathvel@gmail.com
* Correspondence: author. Tel: +886 905605515. E-mail: rudolf.kiefer@tdtu.edu.vn (R. K.)

Citation: Tran, C.B.; Zondaka, Z.; Le, Q.B.; Velmurugan, B.K.; Kiefer, R. Polypyrrole with Phosphor Tungsten Acid and Carbide-Derived Carbon: Change of Solvent in Electropolymerization and Linear Actuation. *Materials* **2021**, *14*, 6302. https://doi.org/10.3390/ma14216302

Academic Editor: Seung Hwan Ko

Received: 27 September 2021
Accepted: 19 October 2021
Published: 22 October 2021

Publisher's Note: MDPI stays neutral with regard to jurisdictional claims in published maps and institutional affiliations.

Copyright: © 2021 by the authors. Licensee MDPI, Basel, Switzerland. This article is an open access article distributed under the terms and conditions of the Creative Commons Attribution (CC BY) license (http://creativecommons.org/licenses/by/4.0/).

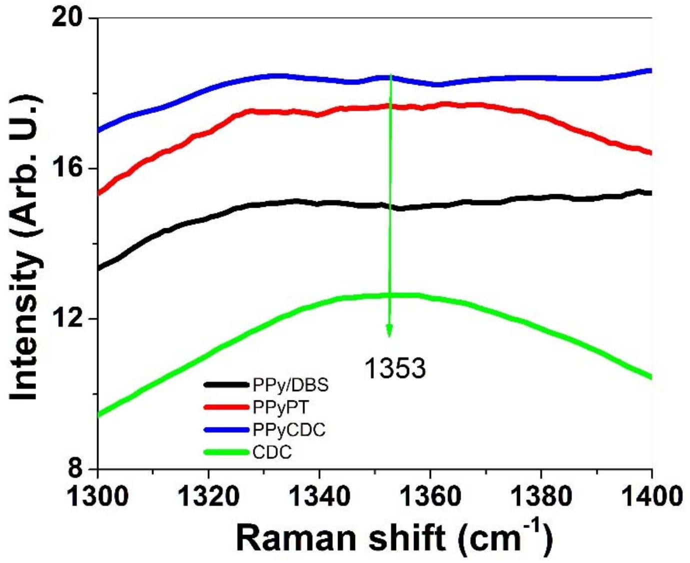

Figure S1. Raman spectra's (1350 – 1450 cm^{-1}, 514 nm) of pristine PPy films in oxidized state (1.0 V) such as PPy/DBS-EG (black line), PPyPT-EG (red line), PPyCDC-EG (blue line) and CDC (green line).

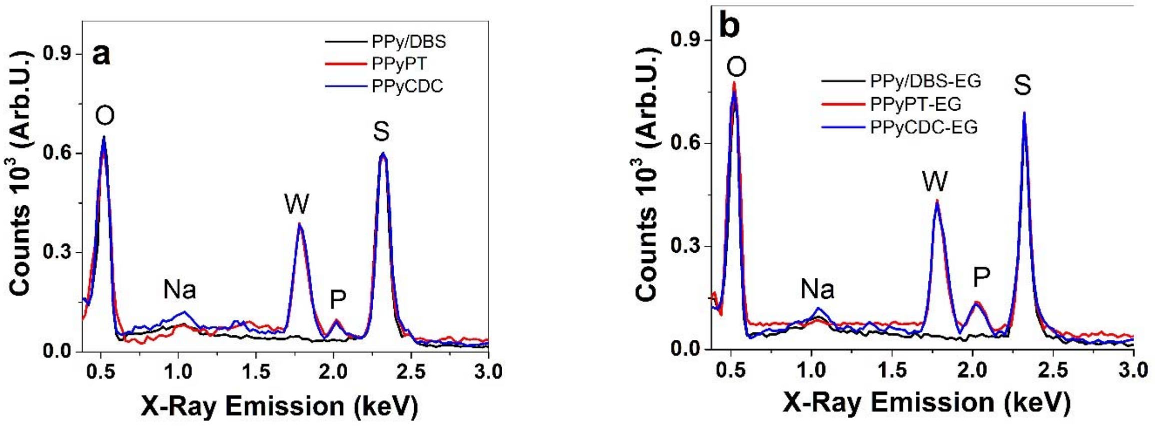

Figure S2. EDX spectroscopy of pristine PPy/DBS and PPy/DBS-EG (black line), PPyPT and PPyPT-EG (red line) and PPyCDC and PPyCDC-EG (blue line) in oxidized state (1.0V) showing in **(a),** PPy composites made in EG:Milli-Q+ (1:1) and in **(b)**, those made in EG solvent.

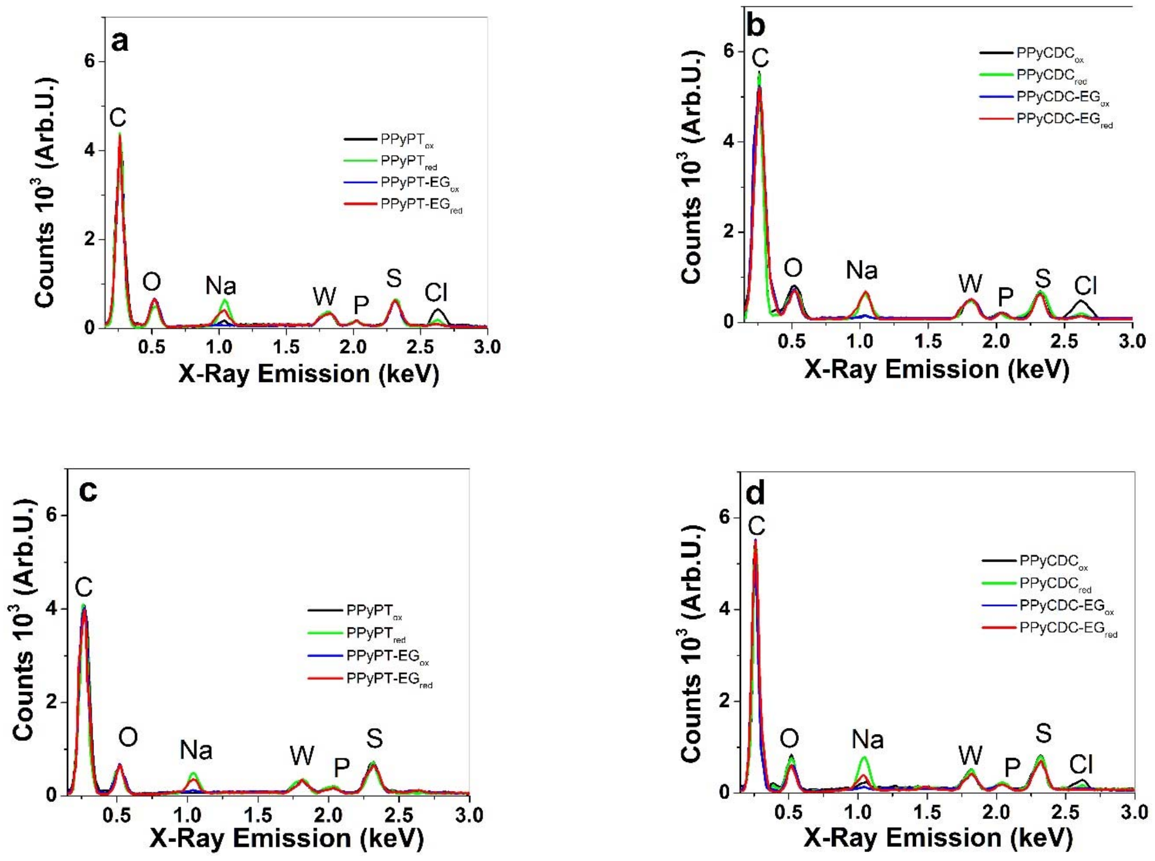

Figure S3. (a), EDX spectroscopy at cross section in oxidized state (5 min, 0.65V) and reduced state (5 min −0.6V) of PPy composite samples such as $PPyPT_{ox}$ and $PPyCDC_{ox}$ (black line), $PPyPT_{red}$, $PPyCDC_{red}$ (green line), $PPyPT\text{-}EG_{ox}$, $PPyCDC\text{-}EG_{ox}$ (blue line) and $PPyPT\text{-}EG_{red}$, $PPyCDC\text{-}EG_{red}$ (red line), in **(b)**, PPy samples in $NaClO_4$-PC and in **(c,d),** those in $NaClO_4$-aq electrolytes.

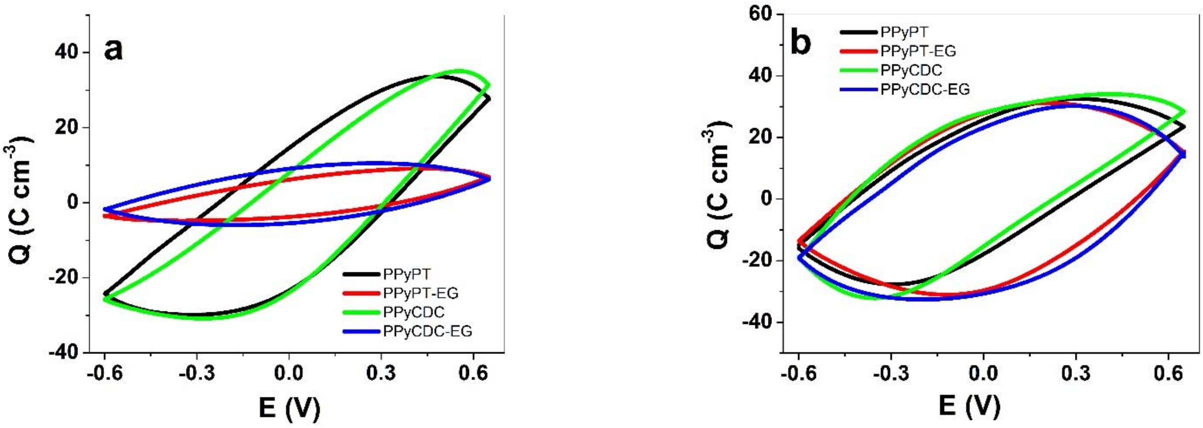

Figure S4. Coulovoltammetry (3rd cycle) of PPyPT (black line), PPyPT-EG (red line), PPyCDC (green line) and PPyCDC-EG (blue line) of charge density against potential E (0.65V to −0.6V) performed in **(a),** of $NaClO_4$-PC and **(b),** $NaClO_4$-aq electrolyte.

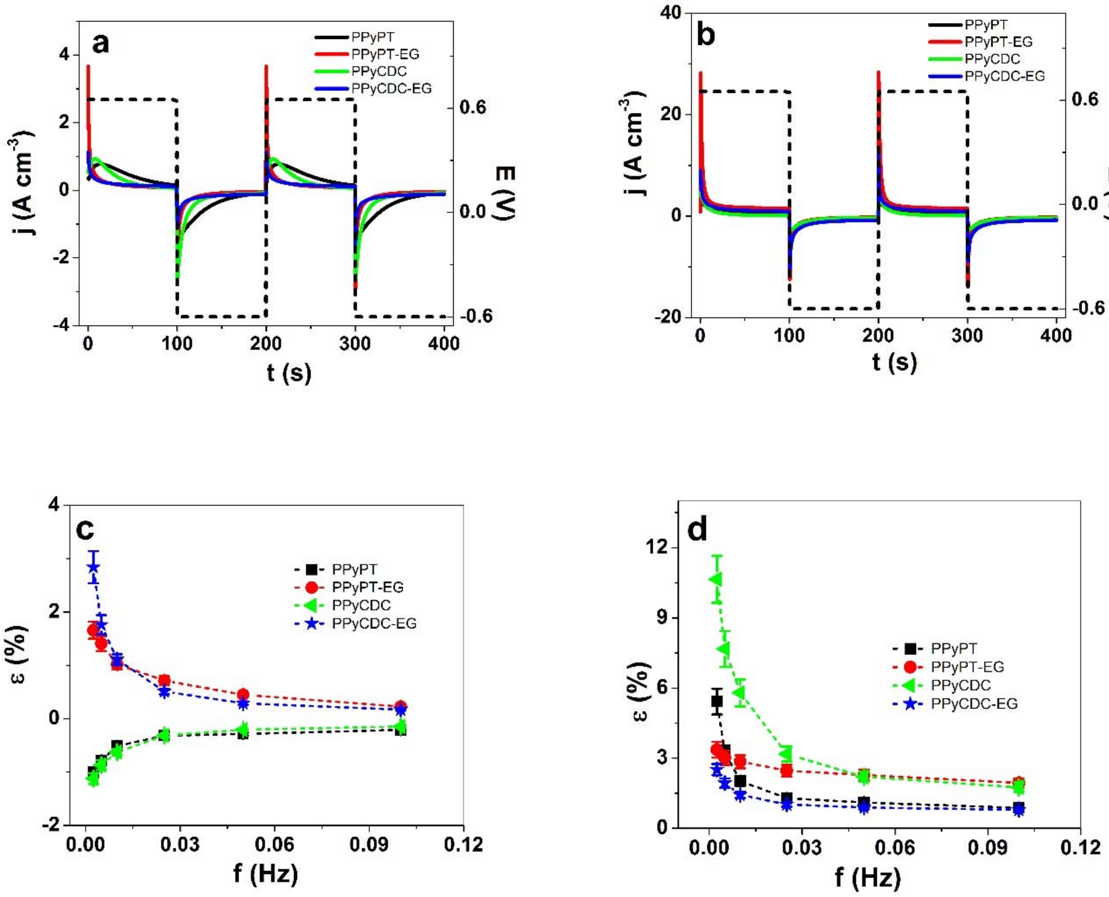

Figure S5. Square potential steps of of PPyPT (black line), PPyPT-EG (red line), PPyCDC (green line) and PPyCDC-EG (blue line) showing current density time curves (two subsequent cyles 2nd to 3rd) at applied frequency 0.005Hz ay potential range 0.65 to −0.6V (E, dashed line) using in **(a),** NaClO4-PC and in **(b),** NaClO4-aq electrolyte. Shown in **(c),** at applied frequencies (f) in range 0.0025Hz to 0.1Hz the PPyPT (··■··), PPyPT-EG (··●··), PPyCDC (··◀··) and PPyCDC-EG (··★··) in $NaClO_4$-PC strain ε and in **(d),** in $NaClO_4$-aq. Negative strain reflecting expansion at oxidation and positive strain expansion at reduction.

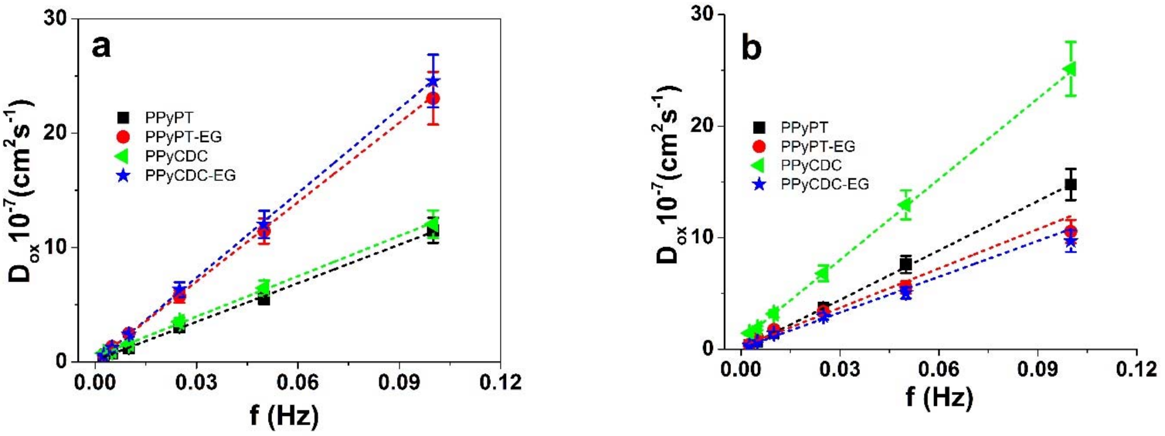

Figure S6. Diffusion coefficients at oxidation D_{ox} of PPyPT (■), PPyPT-EG (●), PPyCDC (◀) and PPyCDC-EG (★) showing in **(a),** $NaClO_4$-PC and **(b),** $NaClO_4$-aq electrolyte against applied frequencies f (0.0025Hz to 0.1Hz). The dashed lines represent the linear fit and shown only for orientation.

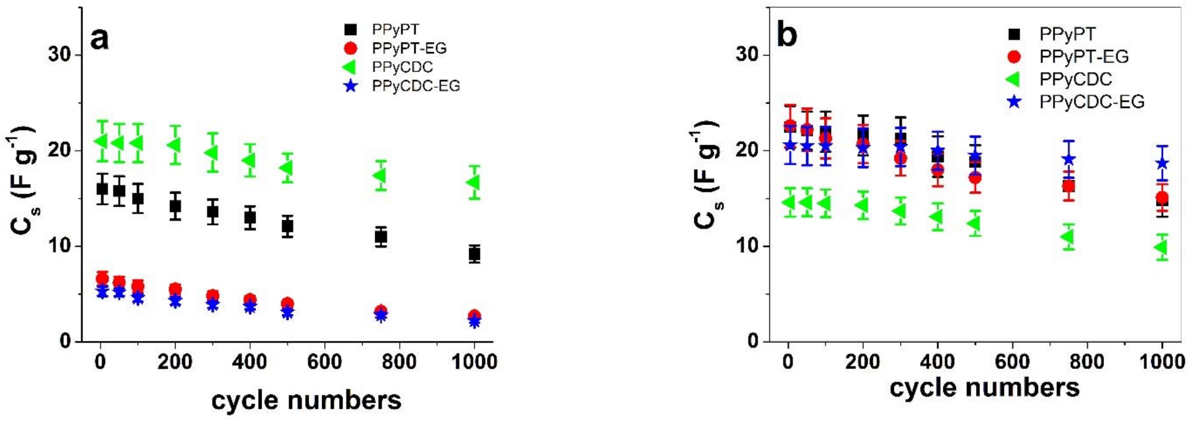

Figure S7. Cycle stability of the specific capacitance C_s against cycle numbers (1000 cycles, 0.1 Hz) at applied current density of ±2 A $g^{-1}$of PPy composites such as PPyPT (■), PPyPT-EG (●), PPyCDC (◀) and PPyCDC-EG (★) applied at different electrolytes showing in **(a),** $NaClO_4$-PC and **(b),** $NaClO_4$-aq.

Table S1. Young's modulus Y of samples such as PPyPT, PPyPT-EG, PPyCDC and PPyCDC-EG from stiffness measurements stretching the film in 1 µm (0.1% strain) to obtain the mass (stress kPa) before and after actuation cycles in $NaClO_4$-PC and $NaClO_4$-aq electrolytes.

**Samples**	**$NaClO_4$-PC**		**$NaClO_4$-aq**	
	**Y [MPa], before**	**Y [MPa], after**	**Y [MPa], before**	**Y, [MPa] after**
PPyPT	115 ± 8.7	55.3 ± 5.7	117 ± 11	88.6 ± 8.2
PPyPT-EG	23.7 ± 2.4	22.1 ± 2.2	14.5 ± 1.2	12.9 ± 1.3
PPyCDC	43.5 ± 4.2	36.3 ± 3.5	21.4 ± 2.1	5.2 ± 0.5
PPyCDC-EG	15.6 ± 1.5	14.5 ± 1.3	21.1 ± 2	19.8 ± 1.7
